# Event and Comment

**Published:** 1906-02-15

**Authors:** 


THE
DENTAL REGISTER.

Vol. LX. February 15, 1906. No. 2.

### EVENT AND COMMENT.

### Porto Rico Dental Law.

The Legislative Assembly of the Island of Porto Rico has enacted a law to regulate the practice of dentistry. There are no new or remarkable features in the law, in fact it copies in general terms the more recently enacted laws for the states and territories. The Board of Examiners is composed of three graduated dentists who have practiced six years in the island, and none of them shall be teachers in a dental college, another case of discrimination against the dental pedagogue. It has often occurred to us that at least one teacher of experience ought to be on every examining board, as he certainly should be able to give good counsel in regard to the extent and quality of the training had by candidates for permission to practice. The meanest feature about the matter is the inference carried that because a man is interested in some dental college that he will not deal honestly and fairly with the candidates. We doubt if dental teachers are more liable to be demoralized than other people. If there is any calling that demands personal integrity it certainly is that of imparting knowledge to the untrained. But we needn't worry about the Porto Rican professors as there are none as yet. The candidate for registration can register for practice on a diploma obtained from a college having a sufficiently high requirement for his diploma, otherwise he must be examined. The board is to determine what schools possess a creditable standard curriculum. The candidate must also have a certificate signed by two freeholders in the town where he expects to locate. The certificate fee is twenty-five dollars.

### The International Dental Journal Discontinues Publication.

The December issue of this journal contains the following notice: "At a meeting of the Directors of the International Dental Publication Co., held October 20th, 1905, it was resolved to discontinue the publication of the *International Dental Journal* with the December issue. Signed, Louis Jack, President." This notice will bring regrets to many who have watched the unequal struggle which this journal has made for principle's sake during the past four or five years. If ever a plucky fight was made this seems to have been one worthy of better success. This journal has had a varied career. It was started in 1880 by Dr. B. M. Wilkinson, the man who invented the dental chair of that name, and a medical man named H. T. Byrd. The journal was published in Baltimore for four years as a medical and dental journal. Dr. Byrd dropped out of the editors place after two years and he was succeeded by Drs. G. H. Rohe and G. W. Field. With the fifth year Dr. W. C. Barrett, of Buffalo, became its editor and a company of New York and Eastern dentists assumed its publication. It then became a dental journal, dropping the medical features. During Dr. Barrett's editorship the journal became one of the most influential journals of the time. The great work that was then being done by Dr. W. D. Miller was presented to American readers through this journal. In 1888 Dr. W. X. Sudduth succeeded Dr. Barrett, and Dr. James Truman succeeded him in 1890, and has continued as its editor until this time.

### The Independent Practitioner.

The journal at first named the "*Independent Practitioner*" and was afterwards changed to "*The International Dental Journal.*" was supposed to stand for the recording of free and independent speech, as distinguished from the prejudiced utterances as recorded in journals whose publishers peddled dental supplies. It is curious to note is passing that its founder never became noted as a professional or literary man, but gave all his time and talent to the development of the chair which bears his name, and we believe he is now or was for many years, wholly employed by one of the greatest dental supply houses in the world. We do not cite this to discredit him or his career; on the contrary, we believe he is worthy of a most honorable place in the list of professional men who have achieved success, for he has by his work done more than many others who are held in great esteem because he has given us the most comfortable and convenient operating chair that ever was made. His dream of establishing a truly independent professional journal was not realized, and it is doubtful if any such undertaking ever will materialize into success. The conditions have never been favorable for such a journal and they are less so now than ever before. We have many excellently edited and published journals now that are printed at the expense of dental supply houses, and all of them are edited with as much independence as though they issued directly from the most strictly professional organization in existence, and no one who will read these journals with an unprejudiced mind will ever suspect that they owe their existence to the trade features which they carry and which in but very few instances are made more conspicuous than the strictly professional matters which are always properly separated. Ours is the oldest dental journal in existence and has, except in the first few years of its existence, had its business affairs conducted by a dental supply house, and in the experience of the present editor, we are most conscientious in saying that we have yet to experience the first interference from our publishers with our etitorial perogatives, and the same has always been so. We have examined ourselves most critically in the ethical mirror for some debasing feature, we confess our eyes are dimmer than they once were, but we can't detect the track of the commercial influence at all. And what is also true we can't find it in other journals working under a similar environment. Where will you find a better or more carefully edited journal than the *Dental Cosmos*? It is now and long has been considered the standard of excellence the world over; and it has probably done as much as any other two dental journals in cultivating professional ideals along with its recording of every important technical and scientific advance that has been made. We could find fault with it, and don't always endorse every pronouncement it makes, but who wants even a dental journal that reflects only his own mind?

We regret exceedingly the failure of the *International Dental Journal*, not that it indicates a low standard of professionalism or loyalty to a principle that needs special or continuous agitation, for its vitalization. This journal has stood for its ideal most loyally and deserves all the credit we can betsow for its strenuous advocacy of an issue, which the profession in general is not willing to discuss or become interested in. The journal was well conducted and recorded some of the best contributions to our professional literature, and if its founders could have seen their way clear to continue its work, we are sure that some day the real benefits that should come from such loyality to a high ideal would have been realized, but perhaps not in the identical form now hoped for. We regret parting with the editor, Dr. Truman, and we trust he may soon find some suitable opportunity for keeping us in touch with his frank and vigorous characterization of the many evil tendencies which threaten our professional existence very much more than the journals published by the dental supply houses.

